# Platelets impair the resolution of inflammation in atherosclerotic plaques in insulin-resistant mice after lipid lowering

**DOI:** 10.1172/jci.insight.193593

**Published:** 2025-10-09

**Authors:** Maria Laskou, Sofie Delbare, Michael Gildea, Ada Weinstock, Vitor De Moura Virginio, Maxwell La Forest, Franziska Krautter, Casey Donahoe, Letizia Amadori, Natalia Eberhardt, Tessa J. Barrett, Chiara Giannarelli, Jeffrey S. Berger, Edward A. Fisher

**Affiliations:** 1 Department of Medicine, Cardiovascular Research Center, New York University Grossman School of Medicine, New York, New York, USA.; 2 School of Medicine, University of Crete, Heraklion, Greece.; 3 Department of Medicine, Section of Genetic Medicine, University of Chicago Pritzker School of Medicine, Chicago, Illinois, USA.

**Keywords:** Cardiology, Inflammation, Vascular biology, Atherosclerosis, Diabetes, Platelets

## Abstract

Insulin resistance impairs benefits of lipid-lowering treatment, as evidenced by higher cardiovascular disease risk in individuals with type 2 diabetes versus those without. Because platelet activity is higher in insulin-resistant patients and promotes atherosclerosis progression, we questioned whether platelets impair inflammation resolution in plaques during lipid lowering. In mice with obesity and insulin resistance, we induced advanced plaques and then implemented lipid lowering to promote atherosclerotic plaque inflammation resolution. Concurrently, mice were treated with either platelet-depleting or control antibodies for 3 weeks. Platelet activation and insulin resistance were unaffected by lipid lowering. Both antibody-treated groups showed reduced plaque macrophages, but plaque cellular and structural composition differed. In platelet-depleted mice, single-cell RNA-seq revealed dampened inflammatory gene expression in plaque macrophages and an expansion of a subset of *Fcgr4^+^* macrophages having features of inflammation-resolving, phagocytic cells. Necrotic core size was smaller and collagen content greater, resembling stable human plaques. Consistent with the mouse results, clinical data showed that patients with lower platelet counts had decreased proinflammatory signaling pathways in circulating nonclassical monocytes after lipid lowering. These findings highlight that platelets hinder inflammation resolution in atherosclerosis during lipid-lowering treatment. Identifying novel platelet-targeted therapies following lipid-lowering treatment in individuals with insulin resistance may be a promising therapeutic approach to promote atherosclerotic plaque inflammation resolution.

## Abstract 2

Platelets impair inflammation resolution in atherosclerotic plaques and interfere with benefits of lipid-lowering in insulin-resistant mice with clinical data consistent with these findings.

## Graphical abstract

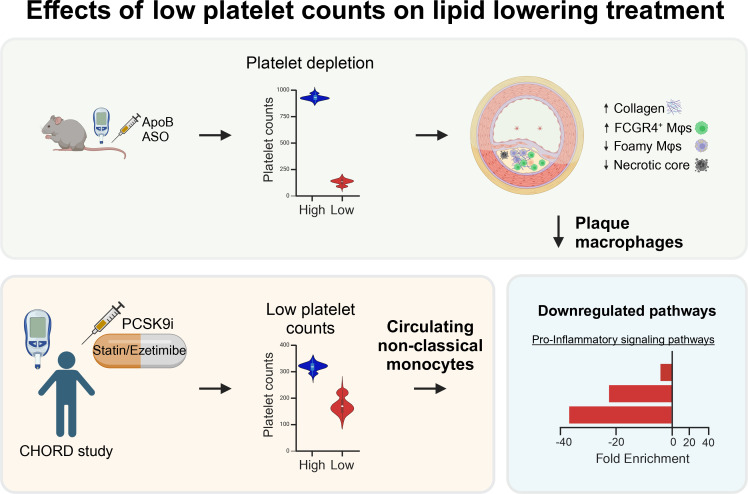

## Introduction

Cardiovascular disease (CVD) remains the leading cause of death in developed nations, a trend expected to persist due to aging of the population and of the growing prevalence of the major risk factors of obesity and type 2 diabetes mellitus (T2D) (1, 2). Indeed, diabesity, encompassing both diabetes and obesity, is increasingly prevalent and strongly associated with atherosclerotic CVD (ASCVD). This is a chronic inflammatory condition (3) characterized by plaque formation within arterial walls, a process that includes lipid accumulation in intimal macrophages (4). Current therapies to reduce ASCVD risk primarily focus on lowering low-density lipoprotein cholesterol (LDL-C) (5). These interventions are successful in halting disease progression, yet risk factors such as the aforementioned obesity, insulin resistance (IR), and diabetes can continue to drive the progression of atherosclerosis and the occurrence of cardiovascular events (6–10).

Another factor that may contribute to this residual risk of ASCVD after LDL-C lowering is the platelet. Platelets are traditionally known for their roles in hemostasis and thrombosis (11), and more recently as mediators of COVID-19–associated cardiovascular complications and mortality (12). Emerging research highlights their involvement in the development and progression of atherosclerosis in mice and people beyond their traditional roles in hemostasis/thrombosis (13–16). Indeed, in a previous study, we have shown that platelets promote an inflammatory state in plaque macrophages during atherosclerosis progression in mice (15). We, therefore, pursued the hypothesis that platelets interfere with the plaque inflammation-resolving benefits of aggressive LDL-C lowering (e.g., see refs. 17–21).

We sought to explore this possibility in a model relevant to the common clinical scenario in which the combination of lipid-lowering therapies with antiplatelet agents is given to patients receiving coronary artery stents (22, 23), many of whom have obesity, IR, or diabetes (23). Thus, we developed a preclinical mouse atherosclerosis model incorporating a dietary protocol to induce obesity and IR/glucose intolerance (24), which we subjected to lipid lowering after establishment of advanced plaques.

In general, reversal of hypercholesterolemia independent of platelet status resulted in several benefits to established plaques, including a decrease in macrophage content. Notably, despite the comparable reductions in macrophage content, platelet deficiency promoted a greater resolution in multiple aspects of plaque inflammation at both the phenotypic level and transcriptional level. To explore the clinical relevance of these findings, we turned to the CHORD study (ClinicalTrials.gov NCT04369664), which focused on people with T2D undergoing aggressive lipid-lowering treatment. Indeed, a lower level of platelets was associated with greater downregulation of proinflammatory pathways in a subset of circulating monocytes correlated with CVD risk (25).

Taken together, the results support a critical role of platelets in the resolution of atherosclerotic plaque inflammation in the insulin-resistant state after lipid lowering. Importantly, the results also demonstrate that beneficial changes in the plaque content of macrophages and in their inflammatory state can be independent of each other.

## Results

### Platelet activation and impaired glucose tolerance are sustained during lipid lowering in atherosclerotic mice.

**Figure 1:**
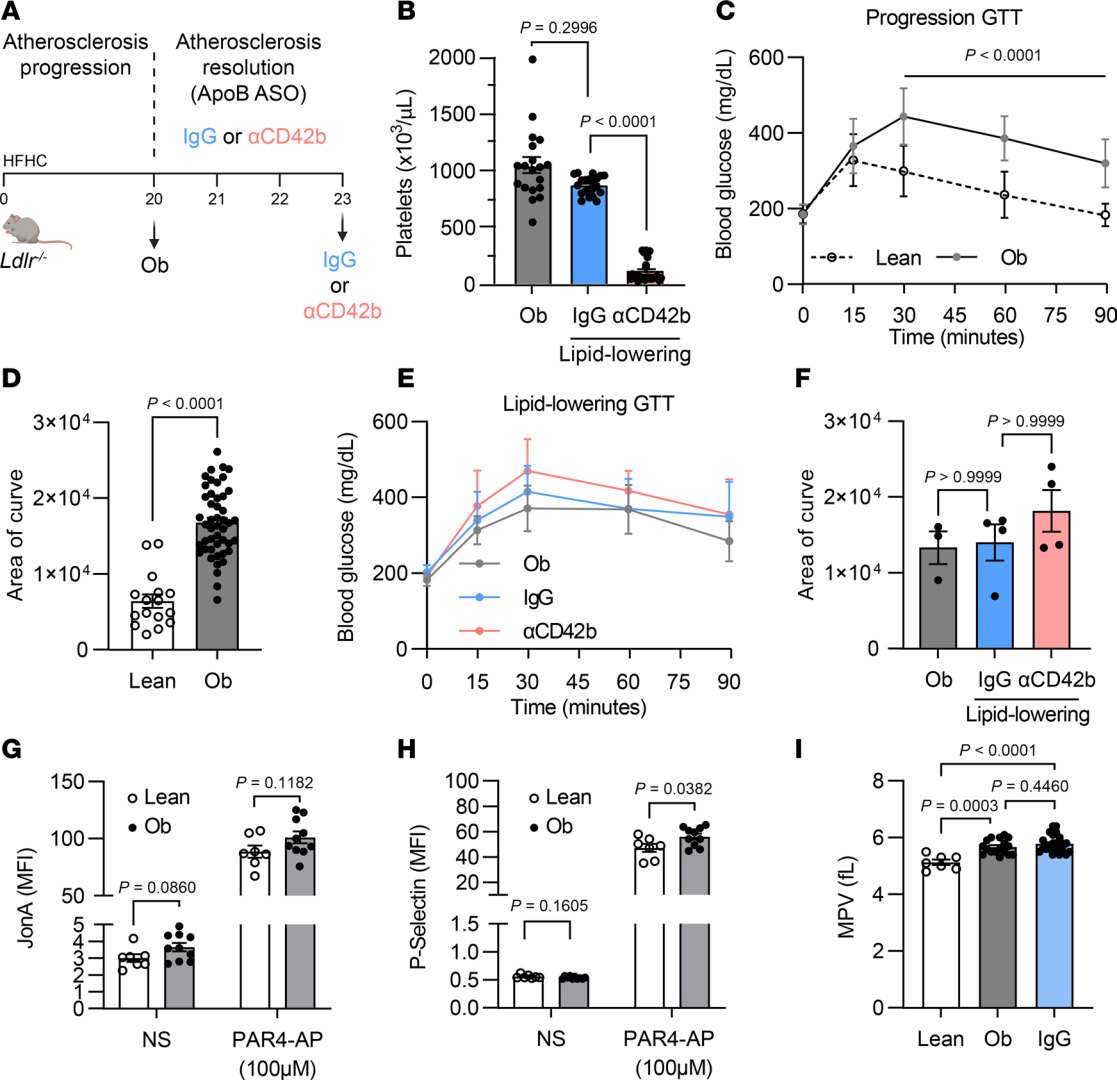
Platelet activation and impaired glucose tolerance are sustained during the lipid-lowering treatment period in obese *Ldlr^–/–^* male mice. (**A**) Study design. Eight-week-old *Ldlr*^–/–^ male mice fed a high-fat, high-cholesterol (HFHC) diet for the whole duration of the study (23 weeks). After the atherosclerosis progression period (obese baseline, Ob), mice were injected with ApoB ASO to lower lipids (lipid-lowering period). All lipid-lowered mice were split into 2 groups, injected either with isotype control antibody (IgG) or platelet depletion antibody (αCD42b) every 3 days for a total of 3 weeks. (**B**) Platelet counts in circulating blood of Ob, IgG, and αCD42b 3 days before harvest. (**C**) Blood glucose measurements (lean *n* = 16, Ob *n* = 47) and (**D**) area of curve (AOC) quantification of glucose tolerance tests (GTTs) from lean, Ob, IgG, and αCD42b mice after 16 weeks of HFHC diet during atherosclerosis progression. (**E**) Blood glucose measurements (Ob *n* = 3, IgG *n* = 4, αCD42b *n* = 4) and (**F**) AOC quantification of GTTs from Ob, IgG, and αCD42b mice after 22 weeks of HFHC diet during atherosclerosis resolution. Mean fluorescent intensity (MFI) of platelet (**G**) JonA and (**H**) P-selectin in nonstimulated (NS) samples and upon 100 μM PAR4-AP agonist stimulation of lean and Ob circulating blood samples. (**I**) Mean platelet volume (MPV) from circulating blood of lean, Ob, and IgG-treated mice, 3 days before harvest. In **C** and **E**, error bars represent SD. Data were analyzed by Kruskal-Wallis with Dunn’s post hoc test (**B** and **F**), unpaired Mann-Whitney test (**C**, **D**, and **H**), unpaired Student’s *t* test (**G**), or ordinary 1-way ANOVA with Tukey’s multiple-comparison test (**I**). *P* values are shown in graphs.

To elucidate the effects of platelets on atherosclerotic plaque inflammation resolution in a setting relevant to clinical obesity and IR, we used a diet-induced obesity model. *Ldlr^–/–^* mice were subjected to a high-fat, high-cholesterol (HFHC) diet for 20 weeks (obese baseline, Ob), which, as expected, induced obesity, hypercholesterolemia (Supplemental Figure 1, A and B; supplemental material available online with this article; https://doi.org/10.1172/jci.insight.193593DS1), and advanced atherosclerotic plaques. After the baseline period, atherosclerotic plaque inflammation resolution was initiated via twice per week administration of an apolipoprotein B (ApoB) antisense oligonucleotide (ASO), which significantly reduced plasma cholesterol levels by suppressing hepatic secretion of the precursor of LDL, namely VLDL (26) (Supplemental Figure 1B). Concurrent with the lipid-lowering treatment, mice received injections every 3 days of either an anti-platelet antibody (αCD42b) we used previously for platelet depletion (15) or an isotype control (IgG) antibody (Figure 1A). Platelet depletion led to a 91% reduction in circulating platelets compared with controls, verified after assessment of circulating platelet counts and platelet marker CD41^+^ cell measurements (Figure 1B and Supplemental Figure 1, C and D). Three weeks of lipid-lowering therapy resulted in equivalent normalization of hypercholesterolemia in both antibody-treated groups (Supplemental Figure 1B).

To evaluate glucose homeostasis during atherosclerosis progression and lipid lowering, we performed glucose tolerance tests (GTTs). Area of curve (AOC) analyses (27) revealed impaired glucose tolerance (iGT) in mice during atherosclerosis progression (Figure 1, C and D), which persisted throughout the lipid-lowering phase independent of the type of antibody treatment (Figure 1, E and F). Additional metabolic parameters were assessed indicative of IR, including elevated fasting glucose and insulin levels (Supplemental Figure 1, E and F), increased the homeostatic model assessment for IR (HOMA-IR) (Supplemental Figure 1G) and reduced quantitative insulin sensitivity check index (QUICKI) measurements (see Methods), which are based on fasting glucose and insulin values (27) (Supplemental Figure 1H) in Ob, IgG, and αCD42b groups compared with lean, age-matched, chow-fed control mice. These findings show that obese mice, with or without lipid-lowering therapy, exhibit sustained iGT and IR, independent of platelet treatment status.

We also measured platelet activity in our Ob and iGT/IR mice during both atherosclerosis progression and lipid lowering. Platelet activation assays in Ob mice demonstrated elevated platelet surface expression of JonA (28) and P-selectin (29) before and after PAR4-AP stimulation (Figure 1, G and H) relative to lean mice. We also measured mean platelet volume (MPV), a crude marker of platelet size, and which is associated with platelet activation (30–32). Compared with lean mice, Ob mice showed an increased MPV, which did not change over time following lipid-lowering treatment (Figure 1I). Thus, platelets remained larger after lipid lowering, suggesting persistent platelet activation. Recently, we found that obese patients with elevated LDL-C have larger and more immature platelets than nonobese patients (33). To validate our findings observed in mice following lipid lowering, we looked at platelet indices in obese versus nonobese patients after lipid-lowering therapy (median LDL-C 31 mg/dL). While platelet counts did not differ between groups, obese patients had larger and more immature platelets (Table 1). Altogether, these murine and human data indicate that the increased platelet activity in obese mice and humans is sustained during lipid lowering.

### Platelet deficiency alters plaque composition after lipid lowering.

Platelets are larger, more prothrombotic, and known to be activated in diabetes as well as in ASCVD (33–35). In preclinical studies they can alter plaque composition, regulate macrophage polarization, and promote atherosclerosis progression (15). Thus, we asked whether platelet depletion influences the ability of lipid lowering to promote atherosclerotic plaque inflammation resolution. Our results revealed that despite no significant changes in plaque size (Supplemental Figure 2A), plaque composition was changed by lipid lowering and platelet depletion.

**Figure 2:**
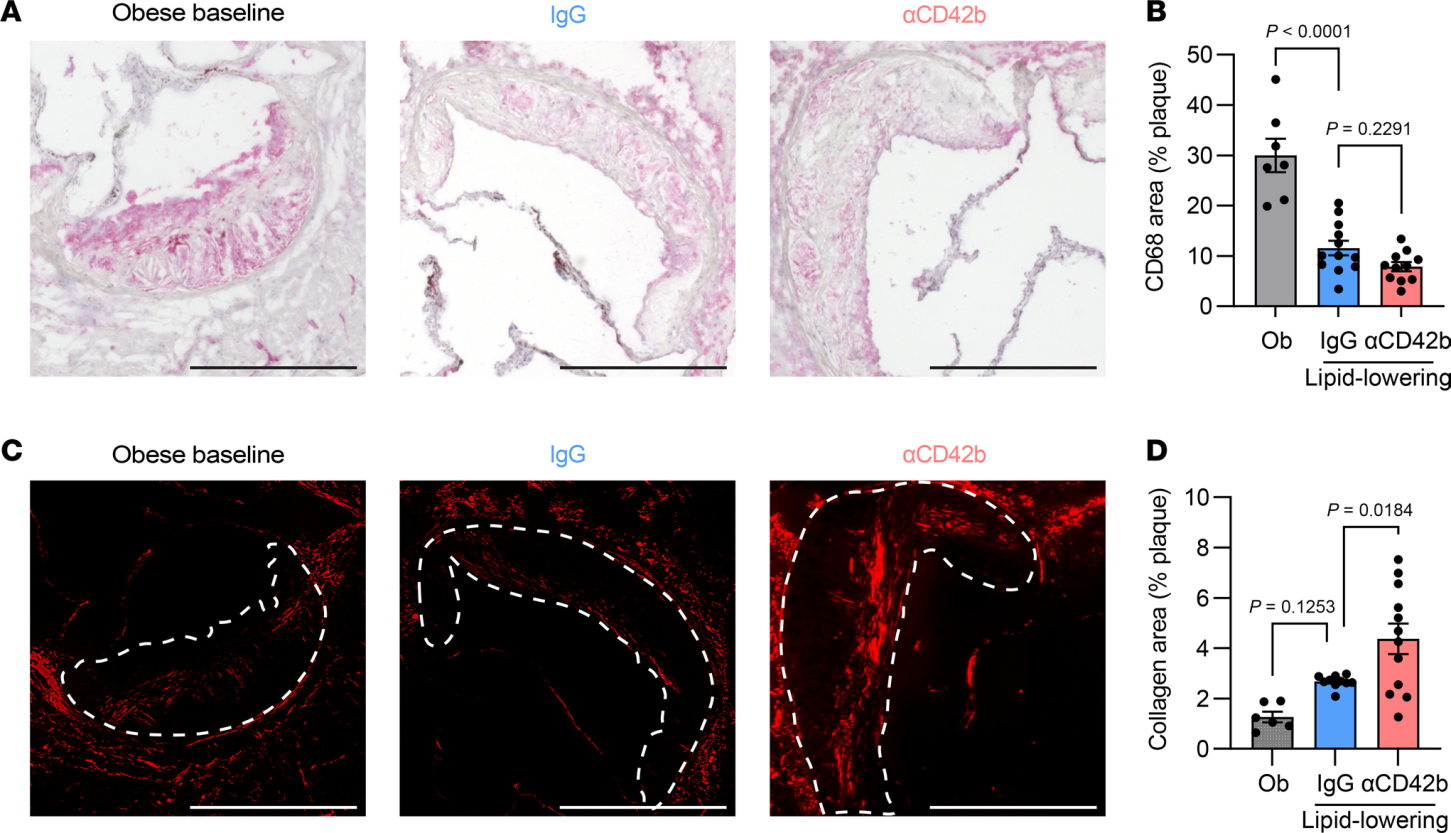
Platelet deficiency alters plaque composition after lipid lowering. (**A**) Representative images (scale bars: 0.5 mm) and (**B**) quantification of immunohistochemical staining for CD68 content (%) in aortic root plaques of Ob, IgG, and αCD42b mice. (**C**) Representative images (scale bars: 0.5 mm) of polarized light and (**D**) quantification of collagen content (%) in aortic root plaques of Ob, IgG, and αCD42b mice. Data in **B** and **D** were analyzed by 1-way ANOVA with Šídák’s post hoc test. *P* values are shown in graphs. Dotted lines in **C** outline the plaques.

In both lipid-lowered groups, there were marked decreases in the percentage of the plaque that was positive for the macrophage marker CD68 compared with Ob (Figure 2, A and B, and Supplemental Figure 2, B and C). The similar plaque content of macrophages in both lipid-lowered groups was consistent, with no significant changes in the recruitment of monocytes into the plaques (Supplemental Figure 2E), as measured by an in vivo trafficking assay (see Methods).

Collagen content of human plaques is an indicator of plaque stability and is thought to be inversely related to the presence of activated macrophages secreting matrix-degrading enzymes (36). It is notable, then, that the percentage plaque collagen content was highest in the platelet-depleted group (Figure 2, C and D, and Supplemental Figure 2D). Consistent with the collagen results, another parameter of clinical plaque stability, the fibrous cap, tended to be of higher area in the platelet-depleted group, as assessed by staining for α-smooth muscle actin (αSMA; Supplemental Figure 2, F and G). These compositional changes in the context of the clinical consensus that plaque composition is an important risk factor for myocardial infarction independent of plaque size (37, 38) highlight the potential clinical relevance of our findings.

Additionally, evidence in the bone marrow (BM) is consistent with no changes between the treatment groups in myeloid progenitors or circulating mature white cells (Supplemental Figure 3, A and B), making these aspects unlikely to be contributing mechanisms to the plaque changes.

### Loss of platelets alters the phenotypes of myeloid cells in atherosclerotic plaques to more inflammation proresolving after lipid lowering.

**Figure 3:**
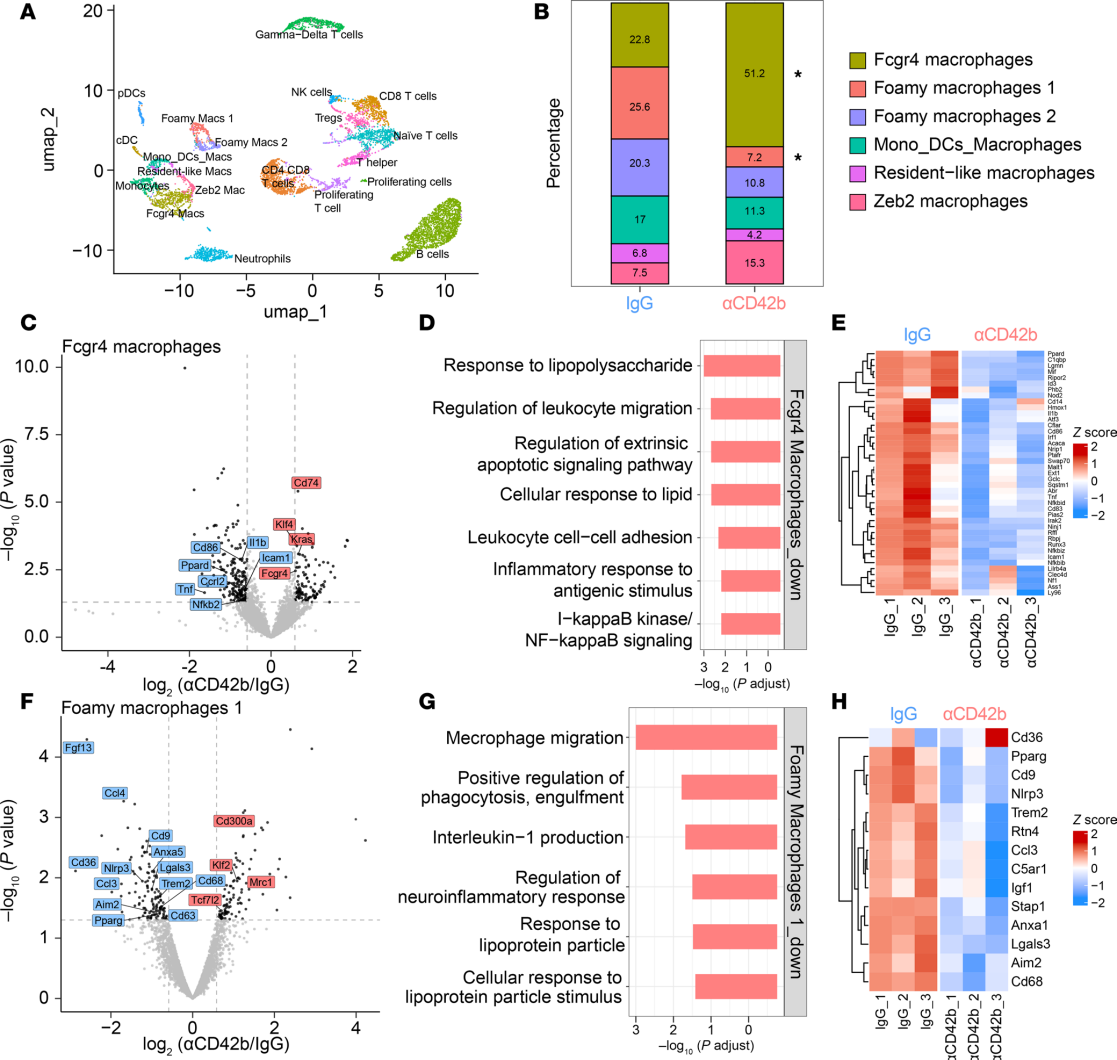
CD45^+^ scRNA-seq shows that loss of platelets changes the phenotype of plaque myeloid cells into a more proresolving state after lipid lowering. (**A**) UMAP embedding of plaque leukocytes showing 20 identified cell types. Μacs, macrophages. (**B**) Percentages of macrophage subtypes identified in IgG and αCD42b macrophages. *P = 0.01 (foamy macrophages), *P = 0.02 (*Fcgr4*^+^ macrophages), using the *t*-test function in the R package propeller. (**C**) Volcano plot showing results of a pseudobulk differential expression analysis of αCD42b relative to IgG samples in the *Fcgr4^+^* macrophage cluster. Significantly differentially expressed genes (DEGs) are highlighted in the plot. (**D**) Overrepresented Gene Ontology (GO) terms (*q* < 0.05) found among significantly downregulated genes in αCD42b relative to IgG samples in *Fcgr4*^+^ macrophage cluster. (**E**) Normalized scaled expression of genes that are associated with overrepresented GO terms shown in **D**. All genes are significantly downregulated in αCD42b-treated mice relative to IgG-treated mice in *Fcgr4*^+^ macrophage cluster. (**F**) Volcano plot showing results of a pseudobulk differential expression analysis of αCD42b relative to IgG samples in foamy macrophage 1 cluster. Significantly DEGs are highlighted in the plot. (**G**) Overrepresented GO terms (*q* < 0.05) found among significantly downregulated genes in αCD42b relative to IgG samples in foamy macrophage 1 cluster. (**H**) Normalized scaled expression of genes that are associated with overrepresented GO terms shown in **G**. All genes are significantly downregulated in αCD42b-treated mice relative to IgG-treated mice in foamy macrophage 1 cluster. In **C**, **E**, **F**, and **H**, *P* < 0.05 and |log_2_(fold change)| ≥ 0.6 were used.

In addition to changes in plaque composition upon lipid lowering and platelet depletion, we wanted to determine whether platelet deficiency alters myeloid cell transcriptome profiles and molecular phenotypes during atherosclerosis resolution. To address this question, we isolated the leukocyte population (CD45^+^ cells) from the plaques. In the subsequent single-cell scRNA-seq (scRNA-seq) analysis, as in other studies (24, 39), we identified multiple subpopulations of leukocytes (Figure 3A), characterized by highly expressed marker genes in each cluster (Supplemental Figure 4A). There were no differences in the proportions of T or B cells (Supplemental Figure 4B). In contrast, our data revealed 2 striking changes in the macrophage populations: a doubling of the macrophage cluster positive for transcripts encoding the low-affinity immunoglobulin gamma Fc region receptor 4 (*Fcgr4*^+^ macrophages) (Figure 3B and Supplemental Figure 4, C and D) and a reduction in the size of the foamy macrophage cluster 1 (Figure 3B) in the platelet-depleted group.

**Figure 4:**
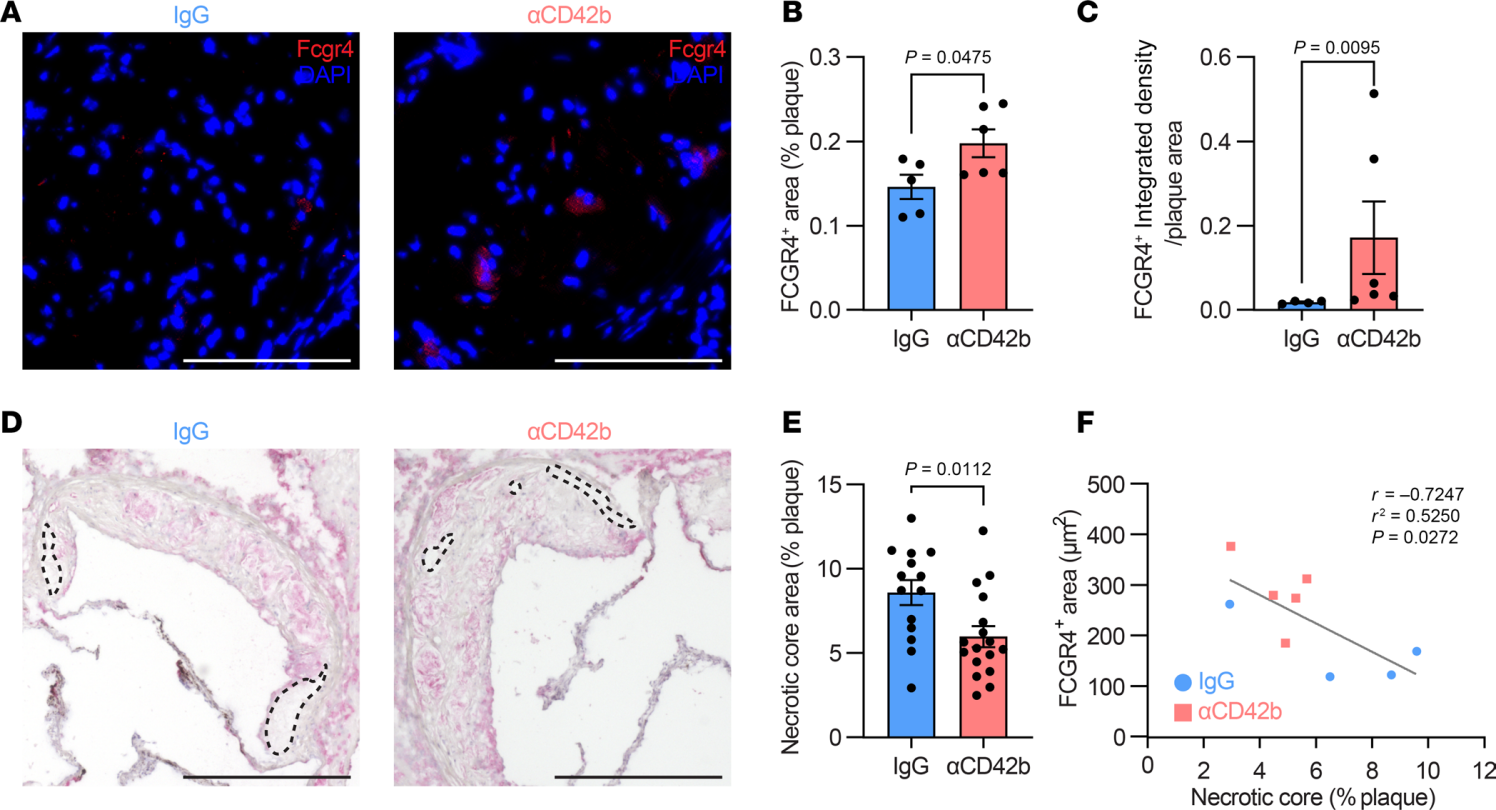
In plaques after lipid lowering, platelet depletion leads to increased abundance of FCGR4^+^ macrophages and reduces necrotic core size. (**A**) Representative images (scale bars: 100 μm) of portions of the aortic root and (**B**) quantification of FCGR4^+^ content (%) in total aortic root plaques of IgG- and αCD42b-treated mice. (**C**) Quantification of normalized FCGR4^+^ integrated density of IgG- and αCD42b-treated mice. (**D**) Representative images (scale bars: 0.5 mm); note that this is the same representative image as shown in Figure 2A, but now with examples of necrotic areas shown (dotted outlines), and (**E**) quantification of necrotic core content (%) of IgG- and αCD42b-treated mice. (**F**) Negative (*r* = –0.7247) correlation analysis between FCGR4^+^ area and necrotic core content from IgG- (blue circles) and αCD42b-treated (pink squares) mice. Data in **B** and **E** were analyzed by an unpaired Student’s *t* test. Data in **C** were analyzed by unpaired Mann-Whitney test. Data in **F** were analyzed by a simple linear regression (*R*^2^ = 0.5250). *P* values shown in graphs.

In other contexts, we reported that *Fcgr4^+^* macrophages (i.e., macrophages with high expression of *Fcgr4*) were associated with inflammation resolution (24, 40). Consistent with this, the transcriptomic analysis of the *Fcgr4^+^* population in platelet-deficient versus -sufficient CD45^+^ cells revealed a downregulation of inflammatory genes (*Tnf*, *Il1b*, *Nfkb2*) and other genes associated with activated macrophages (*Ccrl2*, *Icam1*), as well as upregulation of *Fcgr4* gene expression (Figure 3C). Pathway enrichment analyses of the differentially expressed genes showed a marked downregulation of pathways involved in LPS response, as well as NF-κB and Toll-like receptor (TLR) signaling (Figure 4, D and E, and Supplemental Figure 5, A and B), findings consistent with a reduced inflammatory state.

Gene expression changes in foamy macrophage cluster 1 showed downregulation of foam cell– and lipid-associated transcripts (*Lgals3*, *Trem2*, *Cd9*, *Cd68*, *Cd36*) alongside inflammasome-related genes (*Nlrp3*, *Aim2*) (Figure 3F). Pathway enrichment analysis highlighted a reduction in pathways governing macrophage migration, engulfment regulation, and IL-1 production (Figure 3, G and H).

Together, these data show that after lipid lowering, platelet depletion results in broad changes in gene expression and cellular pathways within specific subpopulations of plaque macrophages, which suggests an adoption of a less inflammatory state.

### Possible mechanisms for the effects of platelets on plaque myeloid cells after lipid lowering.

There are a number of possible mechanisms related to the effects of platelets on the myeloid cell changes we observed in the preceding sections. For one, there is the knowledge that monocyte-platelet aggregates (MPAs) are atherogenic in preclinical models and are associated with increased CVD in patients (15, 41). Comparing circulating MPAs in control IgG- or αCD42b-treated mice, we found that there were significantly decreased numbers in the platelet-depleted (i.e., αCD42b-treated) mice (Supplemental Figure 6A). This relative deficiency of MPAs in platelet-depleted mice, then, could be expected to contribute to some of the additional benefits of lipid lowering in platelet depletion.

Another possibility is suggested by our previous study of atherosclerosis progression (15), in which we reported that in plaque scRNA-seq analyses, platelet mRNA co-isolated with macrophage RNA. These macrophages, enriched in transcripts encoding platelet factor 4 (*Pf4*), suggested either association within the plaques of macrophages and platelets or transport of circulating MPAs into the plaques, with subsequent differentiation of the monocytes into macrophages. We now report that, in the present atherosclerosis inflammation resolution study, there is also a cluster of macrophages in the plaques of IgG control mice that is enriched in *Pf4* (Supplemental Figure 6B).

This could represent a paracrine influence of platelets on macrophages in the plaque. We pursued this idea by analyzing the expression of known cytokine/chemokine/growth factor response genes in macrophages to calculate their activity scores (see Methods). These scores were then used to infer whether macrophages are responding to specific cytokines/chemokines/growth factors in their environment. We identified 31 factors with significantly different activity scores in macrophages between the αCD42b and IgG groups. Of these 31 factors, 9 are known to be expressed, released, or carried by platelets, namely, *FGF2*, *BDNF*, *IL6*, *TGFB1*, *EGF*, *NO*, *HGF*, *CXCL12*, and *TNFA* (Supplemental Figure 6C). These factors, therefore, can be considered as candidate upstream platelet-derived mediators that alter macrophage phenotypes in the plaques.

In our previous studies, IL-6 was found to be a key factor activating macrophages in non-platelet-depleted mice (15). Because *IL6* was identified in the above analysis, we extended this finding to determine whether there is a systemic effect of platelets mediated by IL-6 (as reflected by its levels in the circulation of αCD42b- or IgG-treated mice). The IL-6 ELISA on murine plasma from the 2 lipid-lowered groups, however, showed no significant difference (Supplemental Figure 6D), supporting a more local role for IL-6 and perhaps for other platelet-derived candidate factors.

An additional possibility for the influence of platelets on myeloid cells is that they can be found adherent to endothelial cells (ECs) in atherosclerotic plaques, from where they can secrete cytokines and chemokines affecting leukocyte entry and activation (42–44). Indeed, CD41^+^ staining in atherosclerotic plaques from Ob mice showed platelets attached to the EC layer (Supplemental Figure 6E). Note also that there was also evidence of platelets inside the plaques, consistent with the possibility for the local effects discussed above.

### In regressing plaques, the increased abundance of FCGR4^+^ macrophages with platelet depletion is associated with reduced necrotic core size.

To validate in vivo at the protein level the results from the scRNA-seq analysis (Figure 3B), which showed an increased abundance of *Fcgr4^+^* macrophages in plaques of platelet-depleted mice after lipid lowering, we immunostained aortic root plaques for FCGR4. As shown in Figure 4, A and B, and Supplemental Figure 7A, the area of the plaques positive for FCGR4-expressing cells was significantly increased in the platelet-depleted group. In addition, by image analysis (see Methods), there was also an increased intensity of staining (Figure 4C). Both results are consistent with those at the RNA level (Figure 3, B and C).

Additionally, our previous work (24) has shown an inverse relationship between the plaque content of FCGR4^+^ macrophages and necrotic core size, presumably because of enhanced phagocytic/efferocytotic activity of these cells (also shown in Supplemental Figure 4B) (24). Indeed, analysis of atherosclerotic plaques showed that, in general, the necrotic core area was less in both lipid-lowered groups compared with Ob (Supplemental Figure 7B), consistent with an increased proportion of *Fcgr4^+^* macrophages in both groups relative to Ob (Supplemental Figure 4C). When comparing the lipid-lowered groups to each other, there was an even smaller necrotic core in the plaques from platelet-depleted mice (Figure 4, D and E, and Supplemental Figure 7C), in line with the aforementioned increases in the abundance of FCGR4^+^ cells and the *Fcgr4* expression levels in the platelet-depleted mice (Figure 3C). To show the quantitative relationship between the content of FCGR4^+^ cells and necrotic core, we performed a correlation analysis and found a significant inverse relationship (Figure 4F).

Taken together, these data suggest an important role of platelets in influencing the abundance of FCGR4^+^ macrophages in atherosclerotic plaques, and necrotic core size after lipid reduction.

### The relationship between platelet counts and the phenotype of circulating nonclassical monocytes during lipid-lowering treatment in people with T2D.

**Figure 5:**
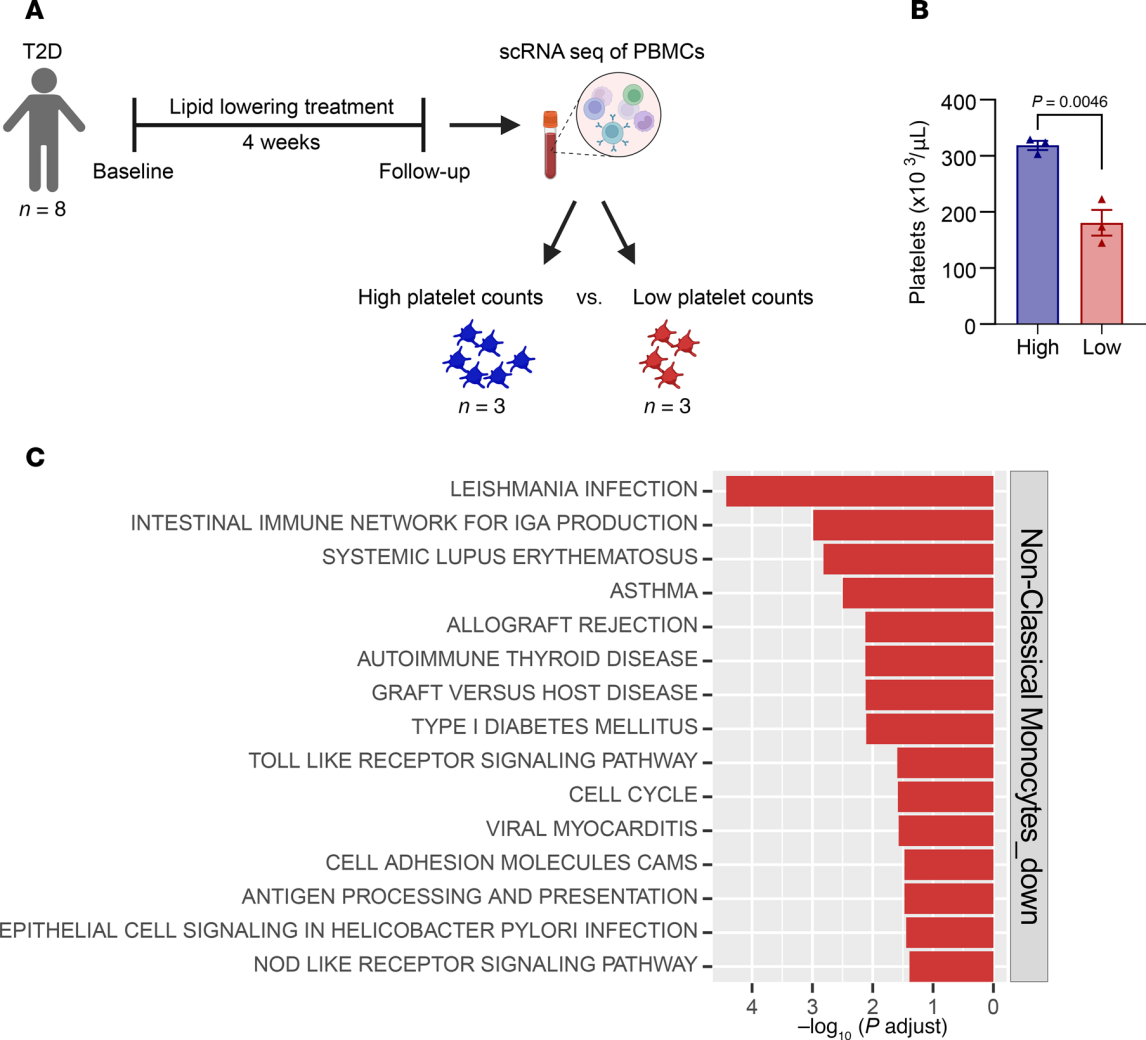
Individuals with T2D and lower platelet counts undergoing lipid-lowering treatment have decreased proinflammatory signaling pathways in circulating nonclassical monocytes. (**A**) CHORD study design. scRNA-seq was performed in PBMCs at follow-up. (**B**) Platelet counts at follow-up from individuals with T2D following lipid-lowering treatments, stratified into the top and bottom third tertile. (**C**) Top 15 downregulated KEGG pathways of nonclassical monocytes in individuals with low (*n* = 3) compared to high (*n* = 3) platelet counts from the CHORD study. Data in **B** were analyzed by an unpaired Student’s *t* test.

To assess the clinical relevance of our findings in humans, we analyzed a scRNA-seq dataset derived from peripheral blood mononuclear cells (PBMCs) collected from a subset of individuals with T2D enrolled in the CHOlesterol Lowering and Residual Risk in Type 2 Diabetes (CHORD) trial (45, 46). Platelet counts did not significantly change during the length of the trial after the administration of an inhibitor of PCSK9 in combination with statin or ezetimibe for 4 weeks (Figure 5A and Supplemental Figure 8A). For this study, we focused on the T2D group with the lowest and highest platelet counts following lipid-lowering therapy, by selecting individuals from the bottom and top tertiles of platelet counts, respectively (Figure 5B).

We were particularly interested in changes in the nonclassical monocytes because of current views that these cells are predisposed to be inflammation resolving once in tissues as macrophages (47). Thus, we hypothesized that nonclassical monocytes would be functionally similar to the *Fcgr4^+^* cluster that we found expanded in the plaques of platelet-deficient mice.

Indeed, pathway analysis of myeloid cell clusters (Supplemental Figure 9, A and B) from individuals belonging to the bottom versus the top tertile of platelet counts following lipid-lowering therapy (Figure 5B) showed significant changes in the inflammatory status of the nonclassical monocyte cluster. Specifically, there was a significant downregulation of proinflammatory pathways, such as TLR signaling, antigen processing and presentation, and cell adhesion pathways in individuals with lower versus higher platelet counts (Figure 5C). These results, although preliminary, are consistent with lower platelet counts having an inflammation-resolving effect on myeloid cells in both humans and mice in insulin-resistant states after lipid lowering.

## Discussion

Platelets play a central role in thrombosis and hemostasis and are recognized as key regulators in atherosclerosis progression, particularly by influencing the macrophage inflammatory state within the plaques (15). This suggests they may counteract the vascular benefits typically associated with LDL-C reduction. Building on our previous study that highlighted proinflammatory effects of platelets on macrophages during atherosclerosis progression (15), we aimed to explore whether platelets interfere with the benefits to plaques of LDL-C lowering, particularly in an insulin-resistant setting, in which it is known that CVD risk is increased (6, 7, 9). By using a clinically relevant mouse model of obesity and T2D/IR, coupled with platelet depletion, we now show that following lipid lowering, (a) platelets, known to be activated during atherosclerosis progression (13–15), remained activated; (b) platelets were associated with sustained inflammatory characteristics of plaque macrophages, but did not affect their content beyond the reduction associated with lipid lowering; (c) scRNA-seq analysis revealed that platelets promoted the expansion of macrophage subclusters with a proinflammatory phenotype and suppressed the frequency of inflammation-resolving and phagocytic FCGR4^+^ macrophages; and (d) platelets adversely affected plaque composition, as reflected by lower collagen content and greater necrotic core area, both signs of instability in human plaques. The clinical relevance of these findings were supported by finding in individuals with T2D after lipid lowering that those with increased platelet counts had upregulated proinflammatory signaling pathways in circulating nonclassical monocytes. Overall, the results suggest that activated platelets, known to be a risk factor for CVD, limit the beneficial effects of lipid lowering to resolve inflammation in atherosclerotic plaques.

Animal models of obesity and diabetes have been essential for understanding how these metabolic disorders affect inflammatory processes within atherosclerotic plaques (10, 24, 48). In the present study, we used a mouse model of diet-induced obesity to promote atherosclerosis progression, hypercholesterolemia, and IR in addition to obesity. Mice continued an HFHC diet during lipid-lowering treatment, reflecting the common poor adherence to lifestyle modifications observed in patients. This also avoided confounding epigenetic changes in macrophages that are dependent on diet composition (48). In addition, it is notable that obese mice on lipid-lowering treatment exhibited sustained iGT and IR.

Platelets have been shown to be hyperactive in obesity, T2D, and atherosclerosis progression (15, 30, 32, 49). Consistent with clinical observations on platelet activation in individuals on statins (50), our data show that platelet activity is sustained in mice undergoing lipid-lowering treatment. Likely contributing to this sustained activation is the persistence of iGT and IR. Based on the present results, this suggests that activated platelets contribute to the challenge of achieving effective atherosclerosis inflammation resolution in diabetic conditions.

Findings by others that also support the adverse effects of platelets on macrophages in plaques include the correlation between platelet counts with plasma IL-1β, a key proinflammatory cytokine regulated by NF-κB signaling (51, 52). Coculture studies also have shown that platelets enhance IL-1β production in macrophages (53). In an atherosclerosis progression study, we previously demonstrated that platelets drive proinflammatory macrophage polarization in atherosclerosis through NF-κB activation, again highlighting their inflammatory role in plaques (15). Related to this, scRNA-seq of plaque CD45^+^ leukocytes from the platelet-deficient mice revealed a reduction in proinflammatory (*Tnf*, *Il1b*, *Nfkb2*) and macrophage activation (*Ccrl2*, *Icam1*) transcripts, along with downregulation of pathways related to LPS response and NF-κB signaling in *Fcgr4^+^* macrophages. Furthermore, bioinformatic analysis indicated that foamy macrophages exhibited lower expression of inflammasome-related transcripts (*Nlrp3*, *Aim2*), highlighting a shift toward a less inflammatory macrophage phenotype in the absence of platelets (Figure 3).

Platelets also have been shown to facilitate oxidized LDL uptake by monocytes and macrophages in vitro (54), a process closely tied to foam cell formation in plaques. Our data align with these findings, demonstrating that foamy macrophages exhibit decreased expression of lipid-associated transcripts (*Lgals3*, *Trem2*, *Cd9*, *Cd68*, *Cd36*) in the absence of platelets during lipid lowering. In this macrophage cluster, differential gene expression revealed downregulated pathways related to engulfment (a mode of lipid uptake), responses to lipoprotein particles, and cellular responses to lipoprotein stimuli (Figure 3).

We also found effects of platelet sufficiency versus deficiency on indices of human plaque stability. For example, by characterizing the plaque composition of platelet-deficient mice after lipid lowering, we observed an increase in collagen content — a positive marker of human plaque stability — without a change in CD68^+^ macrophage content, compared with platelet-sufficient mice (Figure 2). This would be the expected result if plaque macrophages were less activated because of reduced matrix-degrading enzymes.

Another indicator of human plaque stability is the inverse association with the area of the necrotic core. A major negative regulator of the necrotic core area in atherosclerotic plaques is the process of efferocytosis, a form of phagocytosis in which a healthy macrophage takes up a dying cell (55). *Fcgr4^+^* cells are known to have enhanced phagocytic activity (56) and, indeed, our recent work (24) has demonstrated that these macrophages play a crucial role in reducing plaque necrotic core area. This function suggests that *Fcgr4^+^* macrophages contribute to plaque stability through the effective clearance of dying cells, which reduces necrotic core area and inflammatory responses from their damage-associated molecular patterns (DAMPs). It is notable, then, that our scRNA-seq results showed an enrichment of the *Fcgr4^+^* macrophage cluster in platelet depletion over that in platelet-sufficient mice after lipid lowering (Figure 3). Furthermore, the macrophages in the *Fcgr4^+^* cluster in the platelet-depleted mice had an even higher expression of *Fcgr4* mRNA than in platelet-sufficient mice. Quantification of FCGR4^+^ cells in aortic roots of platelet-deficient and platelet-sufficient mice verified the bioinformatic results at the protein level, and a correlation analysis showed a significant inverse relationship between the plaque content of FCGR4^+^ macrophages and necrotic core area (Figure 4), consistent with our previous findings (24). The similar findings in our present and past studies in different models suggest that the macrophage subpopulation characterized by enrichment in *Fcgr4* expression will be a feature of other settings of plaque inflammation.

It should be noted that while the commonly used antibody for platelet depletion in mice (15, 57) provides valuable insights, its use is not directly comparable to clinical antiplatelet treatments like aspirin (58) and clopidogrel (59). Despite this, support for benefits of platelet depletion or inactivation in atherosclerosis independent of the mode of treatment comes from research that has shown that clopidogrel reduces MPAs (59). These are elevated in patients with established CVD (40) and T2D (60), and were reduced in our platelet-depleted mice (Supplemental Figure 6A). In addition, dual antiplatelet and anticoagulant therapy has been shown to inhibit plaque initiation and progression in *Apoe^–/–^* mice (61).

Transcriptomic analysis of PBMCs from the CHORD study enhanced the clinical relevance of the mouse findings by showing that circulating nonclassical monocytes in individuals with T2D at the top tertile of platelets exhibited upregulated proinflammatory pathways, such as TLR signaling, antigen processing/presentation, and cell adhesion pathways (Figure 5). That the human and mouse results are interrelated is supported by cross-species comparison of monocytes (62). Notably, nonclassical (or Ly6C^lo^) monocytes have been shown to be antiinflammatory (63), have atheroprotective properties (64–68), enhanced efferocytosis (69), and benefit tissue remodeling/wound healing (47). These properties resemble those predicted for the *Fcgr4^+^* plaque macrophages. Overall, the human and mouse results suggest that in insulin-resistant conditions, increased platelet counts are a limiting factor to the beneficial effects of lipid-lowering treatments.

In summary, our findings reveal a limiting role of platelets in the inflammation resolution of macrophages in atherosclerotic plaques after lipid lowering in the context of obesity and impaired glucose homeostasis/IR. Importantly, the results show that changes in the macrophage content of plaques can be independent of changes in their phenotypic state, as shown by similar reductions in their number, but very different inflammatory characteristics after lipid lowering in platelet-deficient versus -sufficient mice. In addition, clinical data from individuals with T2D were consistent with these findings. Taken together, these results suggest that targeting platelet activation, function, or abundance during lipid-lowering treatments may offer novel strategies for enhancing plaque stability and reducing CVD risk, particularly in those with IR, such as those with metabolic syndrome and T2D.

## Methods

### Sex as a biological variable.

Male mice have been shown to respond more robustly to diet-induced obesity (70), so only male mice were utilized in this study. Therefore, additional studies are required to explore the potential sex-specific variations in response to platelet depletion.

### Mouse studies.

Male B6.129S7-*Ldlr^tm1Her^*/J mice (*Ldlr*^–/–^; stock 002207, The Jackson Laboratory) were housed in a temperature-controlled (22°C) room on a 12-hour light/dark cycle. For all animal studies, analyses were blinded whenever possible through numerical marking of samples. To power the analyses for atherosclerosis-related endpoints, 8–10 mice per group are sufficient to achieve statistically significant differences (*P* < 0.05) among groups.

Atherosclerosis, obesity, and iGT were induced in 8- to 12-week-old male *Ldlr*^–/–^ mice by HFHC diet feeding (60% fat kcal, 0.3% cholesterol; D17052507, Research Diets) for progression (20 weeks) and lipid-lowering periods (3 weeks). Lipid lowering was achieved by reversal of hypercholesterolemia using an ApoB ASO (26) (Ionis Pharmaceuticals; 50 mg/kg, twice per week for 3 weeks). During the lipid-lowering period, mice were injected every 3 days with 3 μg/g of isotype nonimmune rat IgG control (C301, Emfret Analytics) or to deplete platelets, αCD42b (purified rat monoclonal antibody directed against mouse GPIbα (R300, Emfret Analytics). Mice were assigned to either Ob or 1 of 2 lipid-lowered groups: ApoB-ASO + IgG or ApoB-ASO + αCD42b. Mice were monitored and weighed regularly during the study. For the in vivo analyses and scRNA-seq, mice were excluded if Ob cholesterol levels were less than 600 mg/dL and if lipid-lowered cholesterol levels were greater than 350 mg/dL. Mice were also excluded if they weighed less than 33 g. At the end of the study, mice were euthanized with CO_2_ and blood was collected via cardiac puncture followed by saline perfusion. Aortic roots were embedded in optimal cutting temperature (OCT; Sakura, 4583) and frozen immediately for subsequent sectioning and staining.

### Lipid measurements in mice.

Plasma was isolated by centrifugation. Total cholesterol levels were measured using the Total Cholesterol E Kit (Thermo Fisher Scientific, 99902601) enzymatic assay.

### Platelet counts and flow cytometry in mice.

Circulating blood was collected retro-orbitally 2 days before harvest. Circulating platelet counts were measured in an Element HT5 Heska Hematology Analyzer. Red blood cells were then lysed in Lysis buffer (555899, BD Biosciences) and white blood cells or BM cells were fixed in 2% paraformaldehyde for 10 minutes at room temperature. Cell pellets were washed in Hank’s balanced salt solution (HBSS) with 1% BSA and 1 mM EDTA and stored at 4°C until flow cytometry analysis. Circulating platelets were identified as positive by APC-Cy7 anti–mouse CD41 antibody (133928, BioLegend) and monocytes by PE-Cy7 anti–mouse CD45 (103114, BioLegend) and also PE anti–mouse CD115 positive (135506, BioLegend). MPAs were identified as CD41^+^ cells from CD45^+^CD115^+^ cells. White blood cells and progenitor BM cells were stained and identified as previously described in Scolaro et al. (24).

### GTT in mice.

For GTT, mice were injected intraperitoneally with 2 g/kg body weight of D-glucose (Crystalgen, 300.341.1000) after 6 hours of fasting. Mice had access to water during the experiment. Blood glucose levels were measured before glucose injections (*t* = 0) and again after *t* = 15, 30, 60, and 90 minutes. Blood was collected via tail sampling and measurements were taken using a glucometer (Contour Next EZ, Bayer). Quantification of GTT results was performed by using AOC (27).

### MPV and platelet activity in mice.

MPV was measured in an Element HT5 Heska Hematology Analyzer from retro-orbital blood 2 days before harvest of Ob, IgG, and lean, age-matched, chow-fed baseline control mice. Platelet activation was determined by platelet surface expression of P-selectin and JonA from whole blood flow cytometry, as described previously (15). Briefly, retro-orbital blood from Ob mice was collected 19 weeks after HFHC diet in heparinized capillaries. The whole blood was stained with anti–mouse JonA-PE (M023-2, Emfret Analytics) and anti–mouse P-selectin Alexa Fluor 647 (563674, BD Biosciences) and then treated with PAR4-AP platelet agonist (100 μM; MedChemExpress) for 15 minutes at room temperature. Data were collected on a MACSQuant flow cytometer (Miltenyi Biotec). Gates were established to include platelets. Platelet activation marker JonA or P-selectin mean fluorescent intensity (MFI) was assessed individually.

### HOMA-IR and QUICKI in mice.

For HOMA-IR and QUICKI quantifications, mice were fasted for 4 hours before their glucose measurements were taken with a glucometer. Immediately after, mice were euthanized with CO_2_. Insulin measurements from plasma of fasted mice were performed by using Ultra-Sensitive Mouse Insulin ELISA Kit (90080, Crystal Chem). HOMA-IR and QUICKI calculations were performed as described previously (71).

### Immunohistochemistry and immunofluorescence.

OCT-embedded hearts were sectioned through the aortic root (6 μm) and stained for CD68 (MCA 1957, Bio-Rad) to detect macrophages. Collagen was quantified by measuring the positive areas from the polarized light images (72) after staining of 6-μm aortic root sections with Picrosirius red (PolySciences, 24901-500). Necrotic cores were identified as acellular areas (hematoxylin- and CD68-negative areas) and areas lacking extracellular matrix (which are outlined in dashed black lines in figures), as described previously (24). Briefly, consecutive sections (6 μm apart) of aortic roots were stained with H&E and either Picrosirius red or an antibody against CD68 (PolySciences, 24901-500). The Picrosirius red images were used to confirm the necrotic core area selection in the CD68-stained slides, and then used for quantification. Necrotic core area percentage was quantified as the ratio of the necrotic core area (μm^2^) to the plaque area × 100.

For immunofluorescence, slides were fixed with 4% paraformaldehyde, permeabilized with 0.1% Triton X-100, and blocked with animal serum. Antibody against FCGR4 (50036-T24, SinoBio) was added at a 1:250 dilution for 1 hour at room temperature. Antibody against αSMA (F3777, MilliporeSigma) to stain for vascular smooth muscle cells, antibody against CD41 (CD41-APC, 133914, BioLegend) to stain for platelets, or isotype control (APC Rat IgG1, κ isotype 400412, BioLegend) was added at 1:100 dilution for 1 hour at room temperature.

Sections were then incubated with appropriate secondary antibody (1:400 dilution) and stained with DAPI (P36935, Invitrogen) to detect the nuclei. All images were acquired with a Keyence microscope and analyzed using ImageJ software (73). Integrated density of FCGR4^+^ signal was calculated in ImageJ and normalized to plaque area. Quantification of all staining was performed from at least 5 images per aortic root section per mouse.

### Labeling and tracking of circulating monocytes.

Monocytes were labeled as previously described (15, 74). Briefly, circulating blood monocytes were labeled in vivo by retro-orbital intravenous injection of 1 μm Fluoresbrite YG (17154-10, PolySciences) diluted in 1:4 in sterile PBS, 3 days before harvest. Efficacy assessment was performed in retro-orbitally collected whole blood by flow cytometry 24 hours after injections. All groups were harvested 3 days after injections and labeled monocytes/macrophages were measured as recruited cells in atherosclerotic plaques.

### Aortic arch collection and flow cytometry.

Aortic arches were isolated from all groups after saline perfusion via cardiac puncture. Minced tissue was suspended in digestion buffer containing Liberase (273582, Roche), hyaluronidase (3506, Sigma-Aldrich), and DNase I (DN25, Sigma-Aldrich) in HBSS with 1% BSA and 1 mM EDTA. Tissue was incubated at 37°C for 15 minutes using C-tubes and placed in a gentleMACS dissociator (Miltenyi Biotec), as previously described (24). The digested tissue was filtered through a 100-μm cell strainer, washed with 1× cold PBS, and centrifuged at 350*g* for 5 minutes at 4°C. Aortic arch cells were resuspended in eBioscience Fixable Viability Dye eFluor 660 (65-0864-14, Thermo Fisher Scientific) for dead cell staining, and in PE/Cyanine7 anti–mouse CD45 antibody (103113, BioLegend), which detects all leukocytes. Live CD45^+^ cells were isolated by FACS using a Sony SY3200 highly automated parallel sorting (100 μm) cytometer), for downstream scRNA-seq. In all steps, 0.1 nM flavopiridol (L86-8275, Selleck Chemicals LLC), a broad-spectrum cyclin-dependent kinase inhibitor, was used to arrest cell cycle progression and transcription.

### scRNA-seq of murine plaque cells.

scRNA-seq was performed on live CD45^+^ cells isolated from aortic arches of mice as described above. Cells were then loaded into single-cell gel beads and barcoded with a unique molecular identifier (UMI) using the Single Cell 3′ Reagent kit (10x Genomics, 3′ CellPlex Kit Set A and Cell Multiplexing Oligos; CMO) using the 10x Genomics Chromium iX. Three scRNA-seq libraries were sequenced using an Illumina NovaSeq X+, each with 4 pooled samples from 4 individual mice. Libraries 1 and 2 both contained cells from 1 Ob sample, 1 IgG control sample, and 2 αCD42b-treated samples. Library 3 contained 4 IgG control samples. Cell Ranger v7.1.0 (75) was used to align FASTQ files to the mm10 pre-mRNA assembly, and to obtain UMI and hashtag count matrices. Further processing was done in R v4.2.2 (R Core Team 2021) using Seurat v4.4.0 (76, 77). Samples were demultiplexed and hashtag singlets were retained if they contained 500 or more UMI counts, expressed 250 or more unique genes, and had 20% or less of their reads aligned to mitochondrial genes. DoubletFinder (78) was run on each batch to flag potential doublets. At this point, 2 samples, 1 Ob and 1 αCD42b, were removed from library 1 because of very high levels of erythrocyte contamination (45% and 50%, respectively, of their reads aligned to hemoglobin genes after removal of clusters characterized by high expression of hemoglobin genes; and no clear cluster structure was discernable in their uniform manifold approximation and projection [UMAP] visualization). After quality control, library 1 contained a total of 3,067 cells with on average 4,281 reads per sample; library 2 contained a total of 3,818 cells with on average 6,095 reads per sample; and library 3 contained a total of 812 cells with on average 4,422 reads per sample.

The filtered libraries were normalized, scaled, and corrected for cell cycle scores and percentage reads aligned to hemoglobin genes, using the sc-transform method (79, 80). Integration was performed using Harmony (81). Dimension reduction using UMAP and clustering were performed on the integrated object using the first 20 principal components. T lymphocytes and myeloid cells were further subclustered for a total of 20 cell clusters. Clusters were annotated using known immune cell marker genes (24, 39). For the next set of analyses, the 4 IgG samples in library 3 were merged into 1 sample due to the low number of cells retrieved for each individual sample. The remaining Ob sample was also excluded from downstream analysis, as it did not meet the predefined exclusion criteria. This resulted in 3 IgG controls and 3 αCD42b-treated samples available for downstream analyses. Differences in cell type proportions were tested between αCD42b and IgG using the propeller function in the R package speckle (82), with batch specified as a covariate in the model.

Differentially expressed genes between αCD42b- and IgG-treated samples were identified using a pseudobulk approach in Limma-Voom (83, 84), with batch specified in the model as a covariate. Genes were considered differentially expressed based on a *P*-value cutoff of 0.05 and absolute log_2_(fold change) of 0.6 or greater. Functional enrichment analyses were run using the R package ClusterProfiler (85, 86). Significantly enriched terms were selected using a *q* value of less than 0.05. Significantly enriched GO terms were summarized using rrvgo (87). Figures were made in R using ComplexHeatmap (88) and ggplot2 (89). The numbers of macrophages that were sequenced are comparable between the 2 lipid-lowered groups; no significant differences were observed (number of macrophages in control IgG = 200 ± 42, in αCD42b = 213 ± 38, *P* = 1).

The R package scaper (https://CRAN.R-project.org/package=scaper and https://cran.r-project.org/web/packages/scaper/index.html) was used to calculate cytokine activity scores for each cell, based on gene sets available in the CytoSig database (https://cytosig.ccr.cancer.gov/; Accessed May 2025.). Cytokine activity scores are between 0 and 1 and reflect relative signaling among cells. Cytokine activity scores were compared between IgG- and αCD42b-treated samples by using a Wilcoxon’s test after combining subclusters of macrophages into 1 pool. Wilcoxon’s test *P* values were corrected for multiple testing.

### Participants enrolled in the CHORD study.

Individuals with T2D were enrolled in the CHORD study (ClinicalTrials.gov NCT04369664), as previously reported (45, 46). Participants were administered cholesterol-lowering medicines, which included a PCSK9 inhibitor and statin or ezetimibe for 1 month. Platelet counts were measured at baseline and 4 weeks later, at follow-up. Participants were categorized based on their platelet counts at follow-up. Obesity was defined as BMI greater than 30.

### scRNA-seq of human PBMCs.

scRNA-seq data from PBMCs had been previously published by Barcia Durán et al. (45) and made publicly available (NCBI GEO GSE272294). Data were downloaded from the GEO and processed following the methods detailed in GitHub (https://github.com/giannarelli-lab/Immune-checkpoint-landscape-of-human-atherosclerosis-and-influence-of-cardiometabolic-factors). The data were processed with the Seurat package (v.4.4.0) within R (v.4.1.2). Nonclassical monocytes from high- and low-platelet samples described above were isolated. Differential expression between high- and low-platelet samples was performed using Wilcoxon’s rank-sum test via RunPresto within the SeuratWrappers package (v.0.3.1) with logfc.threshold = 0, min.cells.group = 1, and min.pct = 0.1. Genes with an FDR-adjusted *P* value of less than 0.1 and a log_2_(fold change) greater than 0.5 or less than –0.5 were considered differentially expressed. For pathway enrichment, KEGG pathway annotations were accessed through msigdb using the msigdbr package v7.5.1 (https://cran.r-project.org/web/packages/msigdbr/). KEGG pathway over-enrichment analysis was performed on the differentially expressed genes using enrichR package v3.4 (https://cran.r-project.org/web/packages/enrichR/) function with the universe parameter set to all genes in the RunPresto output. Pathways with an FDR-adjusted *P* value of less than 0.1 were considered significantly enriched.

### Statistics.

Prism 9 (GraphPad Software) was used for in vivo experiment analyses. Data are presented as mean ± SEM unless otherwise indicated. Data with *P* values of 0.05 or less were considered statistically significant. All data were tested for outliers by robust regression and outlier removal and for normality and lognormality by Shapiro-Wilk test. Data that were determined to be parametric were analyzed by a 2-tailed unpaired Student’s *t* test (2 groups) or 1-way ANOVA (>2 groups) followed by Šídák’s, Dunnett’s, or Tukey’s multiple-comparison test, as noted in the figure legends. Data that were determined to be nonparametric were analyzed by unpaired Mann-Whitney (2 groups) or Kruskal-Wallis (>2 groups) followed by Dunn’s multiple-comparison test, as noted in figure legends. For the CHORD study, data were analyzed by a 2-tailed unpaired or paired Student’s *t* test, as noted in figure legends.

### Study approval.

Mice: All experimental procedures were done in accordance with the US Department of Agriculture Animal Welfare Act and the NIH *Guide for the Care and Use of Laboratory Animals* (National Academies Press, 2011), and they were approved by the New York University (NYU) School of Medicine’s Institutional Animal Care and Use Committee (protocol number IA16-00494). The human study was approved by the Institutional Review Board (IRB) of NYU Langone Health (IRB no. 21-00429). Written informed consent was received by individuals prior to participation.

### Data availability.

scRNA-seq data of plaque macrophages are publicly available in the NCBI GEO with accession number GSE286091. scRNA-seq data of PBMCs had been previously published (45) and made publicly available in the GEO with accession number GSE272294. All data in figures are reported in the Supporting Data Values file.

### Artificial intelligence technologies.

ChatGPT 4.0 was utilized as a writing assistance tool in the preparation of this manuscript to improve the grammar and correct typographical errors in the first author’s (ML) draft. The manuscript was also completely edited by the corresponding author (EAF) and the other authors.

## Author contributions

ML and VDMV performed mouse experiments, data collection, and data analysis. SD performed the bioinformatic analysis for murine samples. AW, MLF, FK, and CD assisted with data collection. LA and NE processed human samples and performed the scRNA-seq. MG performed the bioinformatic analysis for human samples. JSB conducted the CHORD study and provided human PBMCs. CG provided resources for sequencing human samples from the CHORD study. ML, AW, MLF, FK, TJB, JSB, and EAF provided intellectual input. EAF provided resources, supervision, and contributed to the design of research. ML and EAF wrote the manuscript. ML, SD, AW, LA, CD, TJB, CG, JSB, and EAF edited and revised the manuscript. All authors approved the final submission.

## Funding support

This work is the result of NIH funding, in whole or in part, and is subject to the NIH Public Access Policy. Through acceptance of this federal funding, the NIH has been given a right to make the work publicly available in PubMed Central.

- American Heart Association grant AHA-SFRN 20SFRN35210936 (to EAF, JSB, and CG).
- NIH grant R35HL144993 (to JSB).
- NIH grants R01HL153712 and R01HL165258 (to CG).
- PolyBio Research Foundation (to CG).
- RECOVER grant ROA-OTA-21-015 (to CG).
- NIH grant R01HL167917 (to TJB).
- Harold S. Geneen Charitable Trust (to AW).
- NIH grants HL151963 and HL131481 (to AW).
- German Research Foundation (DFG) fellowship 39828454 (to FK).
- NIH Cancer Center Support Grant P30CA016087 (to NYU Langone’s Genome Technology Center [RRID: SCR_017929]).
- NIH Cancer Center Support Grant P30CA016087 (to the Laura and Isaac Perlmutter Cancer Center), which partially supports NYU Langone’s Genome Technology Center.

## Supplementary Material

Supplemental data

Supporting data values

## Figures and Tables

**Table 1:**
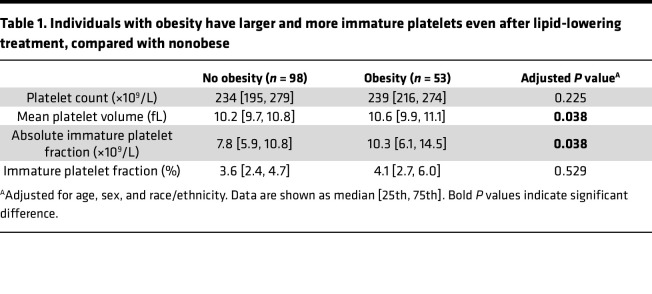
Individuals with obesity have larger and more immature platelets even after lipid-lowering treatment, compared with nonobese

## Version 1 10/09/2025

In-Press Preview

## Version 2 11/10/2025

Electronic publication
