# Supplementary material for: Platelets impair the resolution of inflammation in atherosclerotic plaques in insulin-resistant mice after lipid lowering
Source: JCI Insight. 2025 Oct 9;10(21):e193593. doi: 10.1172/jci.insight.193593 (PMC12643486; doi:10.1172/jci.insight.193593)
Supplement: Supplemental data [file jciinsight-10-193593-s040.pdf]

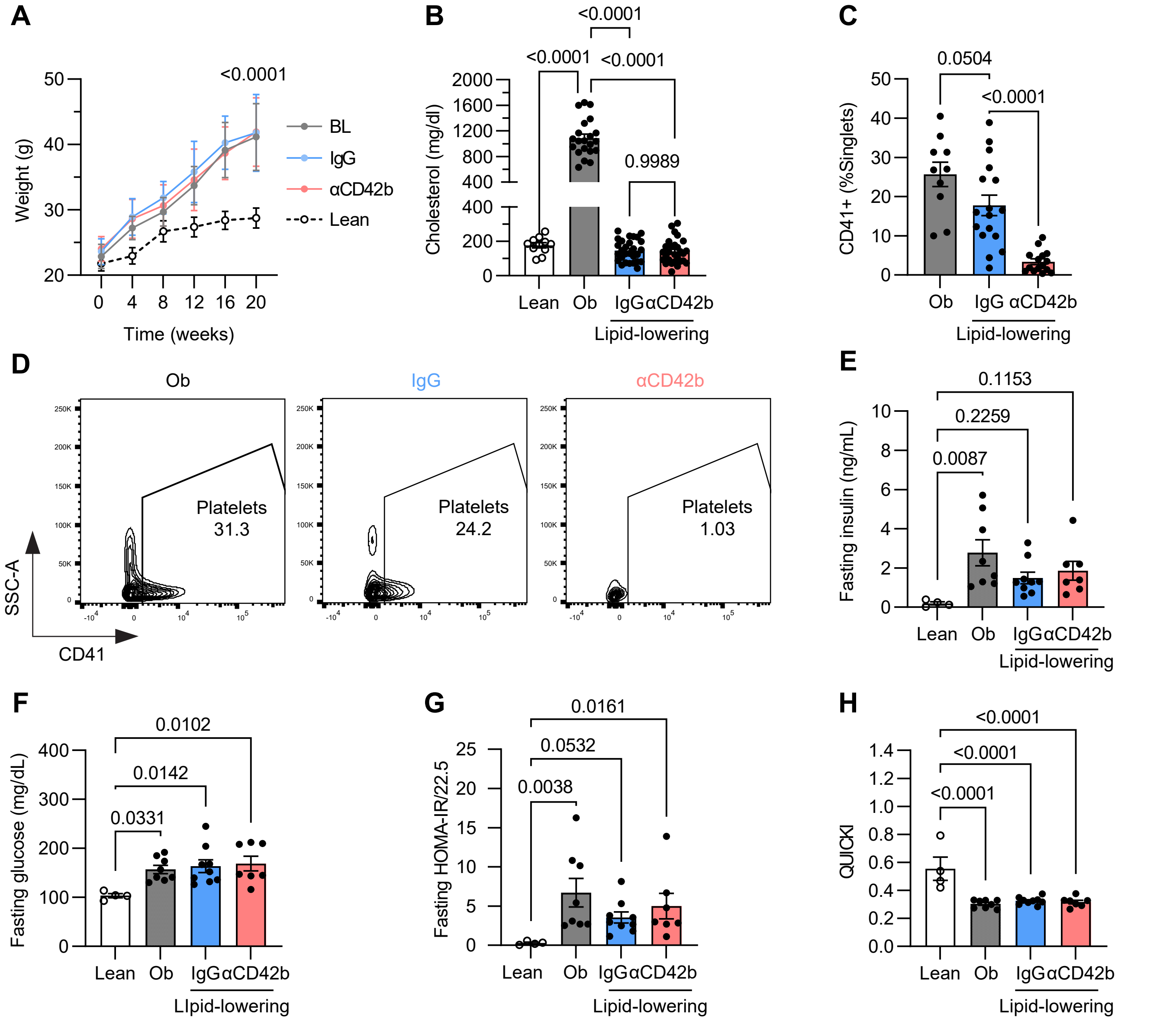

Supplemental Figure 1. Impaired glucose tolerance and platelet activation are sustained during the lipid-lowering period in diet-induced obese and atherosclerotic *Ldlr*$^{-/-}$ male mice. (**A**) Weights (Lean n=14, Ob n=13, IgG n=17, αCD42b n=17) and (**B**) plasma cholesterol levels of Lean, Ob, IgG, αCD42b mice at harvest. (**C**) Gating strategy and (**D**) quantification of CD41+ cells in circulating blood of Ob, IgG and αCD42b mice three days before harvest. (**E**) Fasting glucose and (**F**) fasting insulin levels. (**G**) HOMA-IR calculations and (**H**) QUICKI quantification of Lean, Ob, IgG, αCD42b mice. In **A**, error bars represent SDs. Data in **A** and **B** were analyzed by Two-way ANOVA Dunnett's post hoc and One-way ANOVA Tukey's post hoc test, respectively. Data in **D** were analyzed by One-way ANOVA Sidak's post hoc test. Data in **E**, **F** and **H** were analyzed by One-way ANOVA Dunnett's post hoc test. Data in **G** were analyzed by One-way ANOVA Dunn's post hoc test. p-values are shown in graphs.

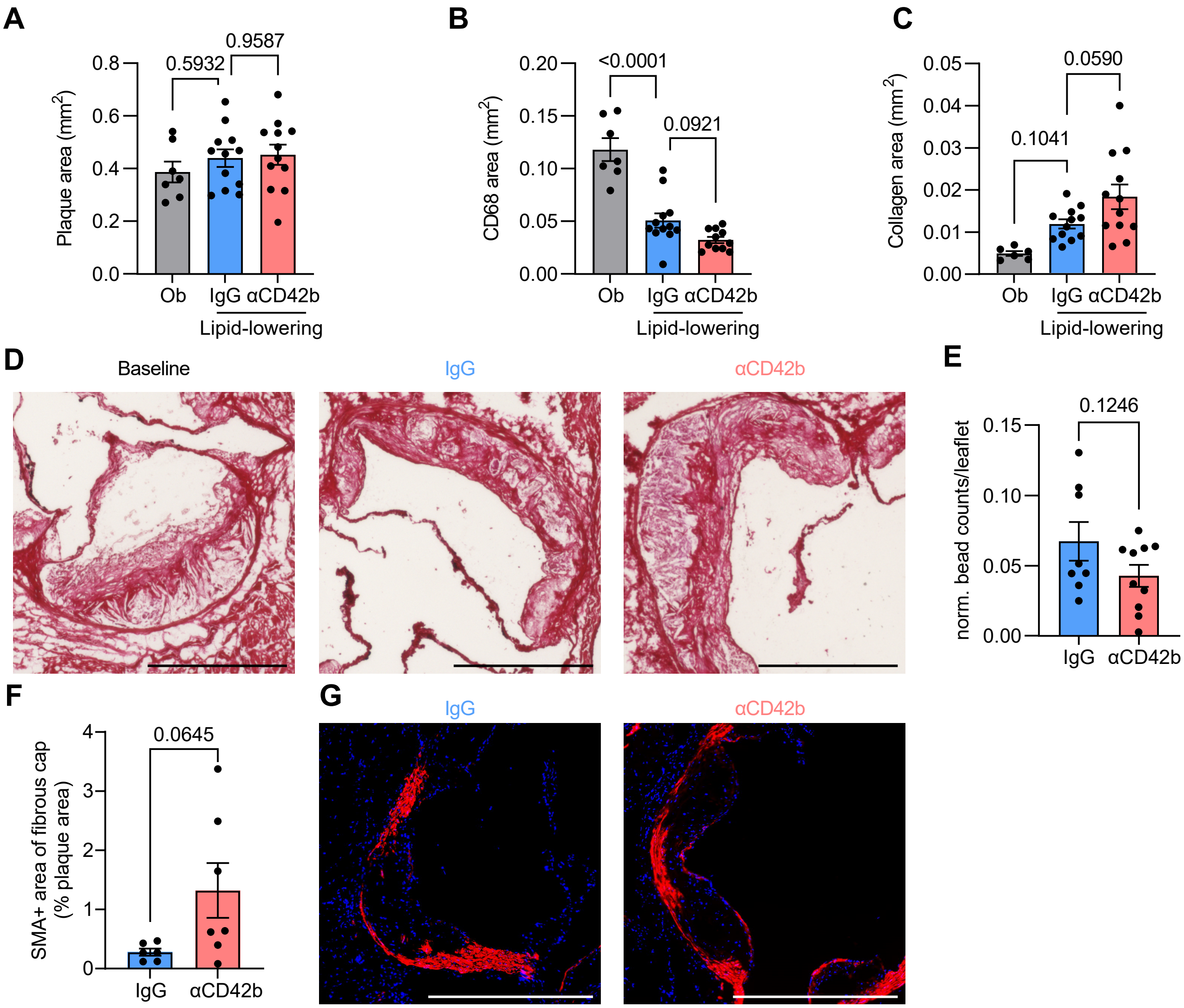

Supplemental Figure 2. Platelet deficiency alters plaque composition after lipid-lowering. (**A**) Quantification of plaque burden (mm^2) of aortic roots from Ob, IgG- and αCD42b-treated mice. (**B**) Quantification of immunohistochemical staining for CD68 area (mm²) in aortic root plaques of Ob, IgG- and αCD42b-treated mice. (**C**) Quantification of collagen area (mm²) in aortic root plaques of Ob, IgG- and αCD42b-treated mice and (**D**) brightfield representative images (scale bar=0.5mm). (**E**) Normalized bead counts of monocytes recruited in aortic root plaques of IgG- and aCD42-treated mice. (**F**) Representative images and (**G**) quantification of SMA^+ area of fibrous cap from IgG- or αCD42b-injected mice. Data in **A**, **B**, **C** and **E, F** were analyzed by One-way ANOVA Sidak's post hoc test and unpaired Student t-test, respectively. p-values shown in graphs.

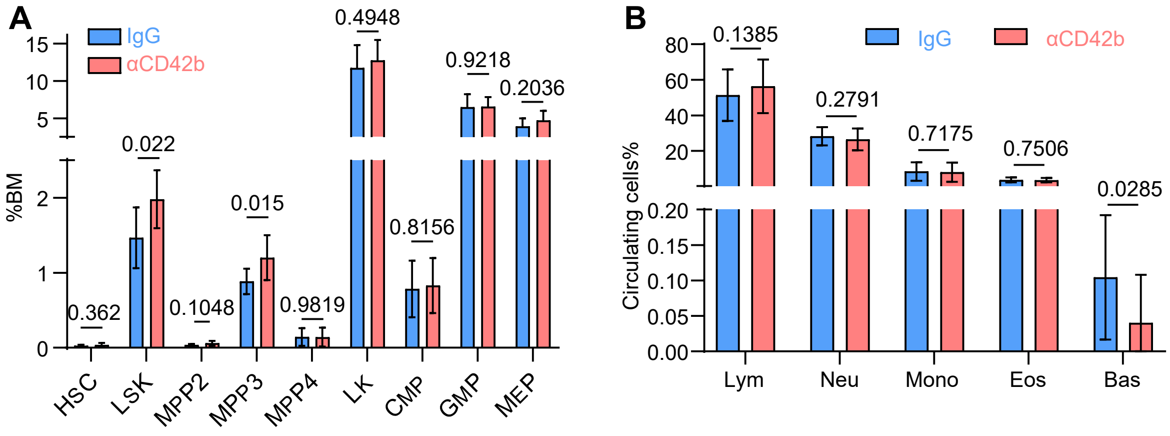

Supplemental Figure 3. Circulatory white blood cells and bone marrow progenitors in IgG- and αCD42b-treated mice. (**A**) Bone marrow progenitor cells and (**B**) white blood cells in circulation from IgG and αCD42b-treated mice. Data in **A** and **B** were analyzed by unpaired Mann Whitney. p-values shown in graphs.

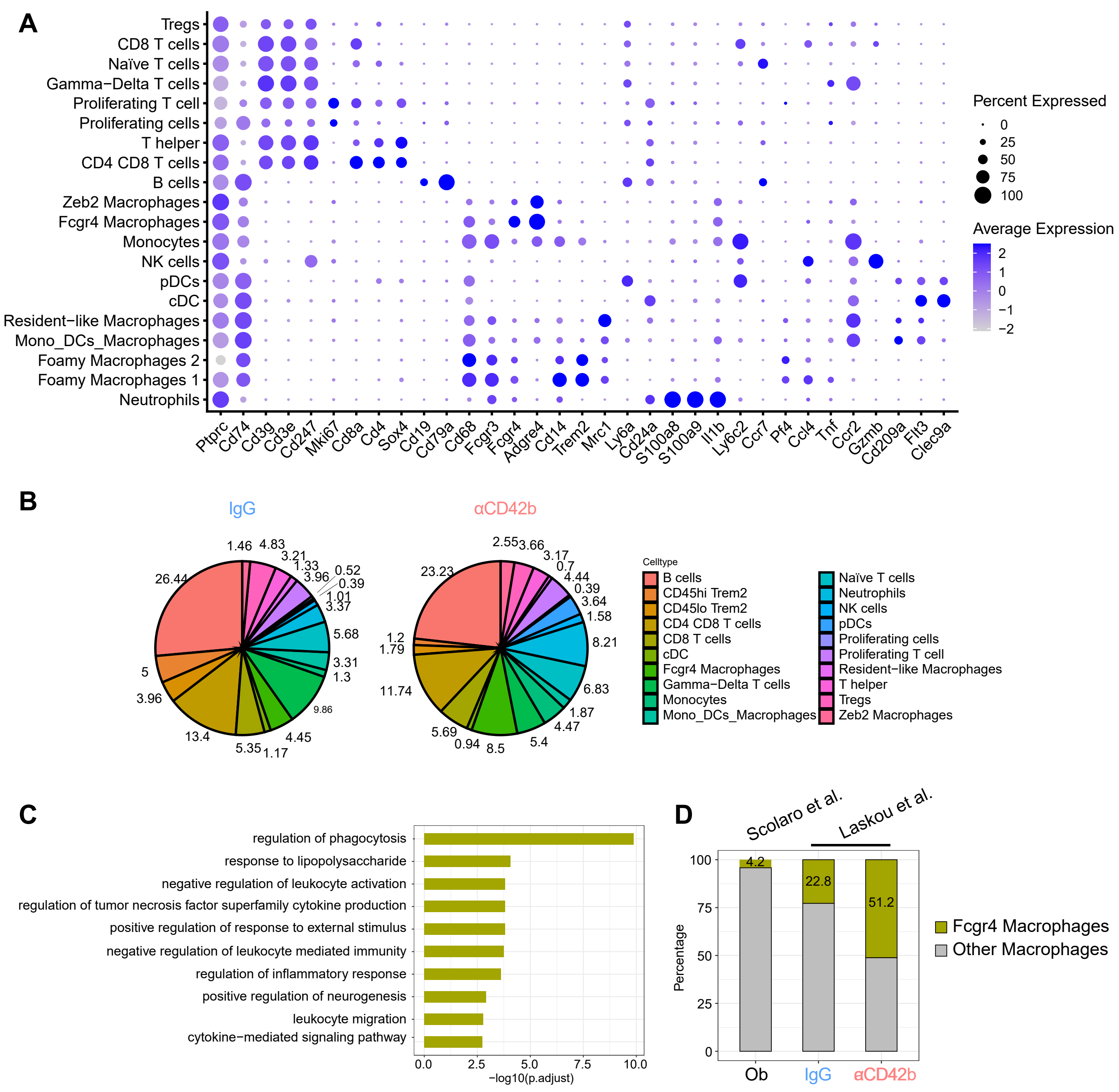

Supplemental Figure 4. CD45⁺ scRNA seq of platelet depleted mice reveals an increase in *Fcgr4*⁺ phagocytic macrophages and downregulation of inflammatory pathways after atherosclerosis inflammation resolution. (**A**) Heatmap showing normalized scaled expression of marker genes used to identify 20 cell types in leukocyte single-cell RNA-seq. (**B**) Proportion analysis of all clusters. (**C**) Top 10 most significantly overrepresented GO terms found among the top 100 marker genes of *Fcgr4*⁺ macrophage cluster. (**D**) Percentage of *Fcgr4*⁺ macrophages identified among all macrophages in αCD42b-treated mice, IgG-treated mice, and baseline obese (Ob) mice obtained from Scolaro et al. (24).

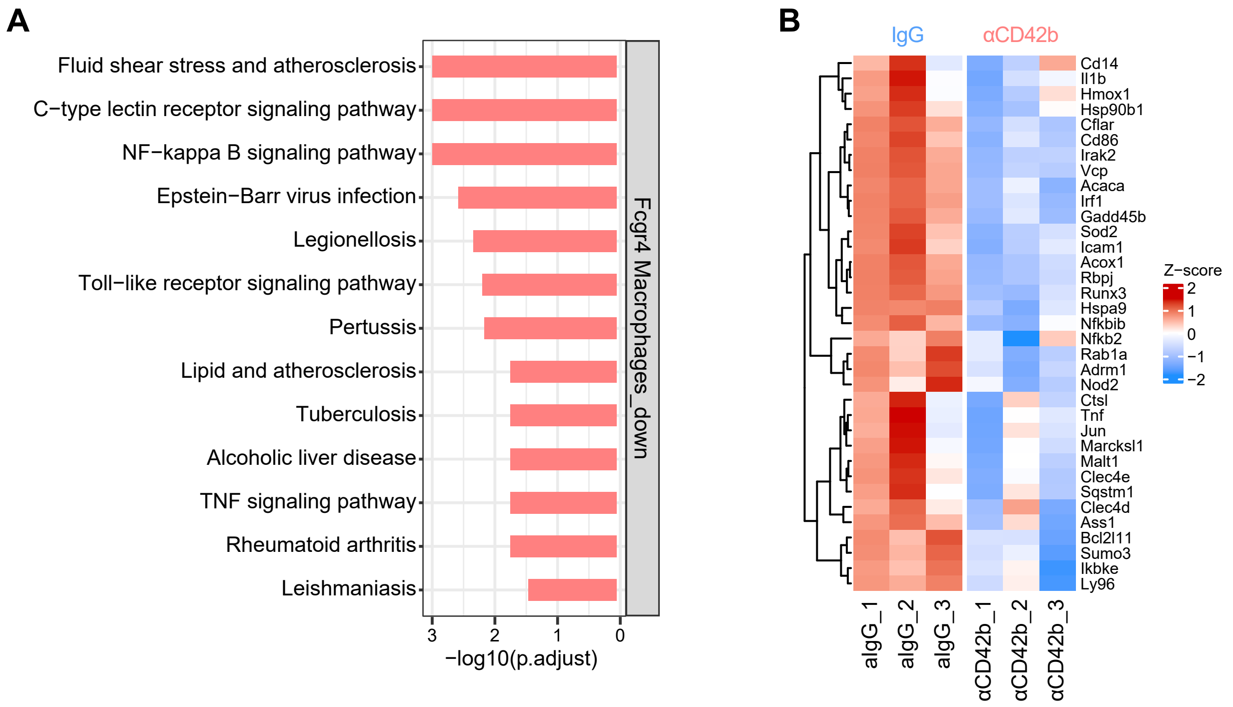

Supplemental Figure 5. CD45⁺ scRNA seq of platelet depleted mice reveals a downregulation of inflammatory pathways in *Fcgr4*⁺ macrophages during lipid-lowering. (**A**) Overrepresented KEGG terms (q-value<0.05) found among significantly downregulated genes in α CD42b relative to IgG samples in *Fcgr4*⁺ macrophage cluster. (**B**) Normalized scaled expression of genes that are associated with overrepresented KEGG terms shown in Suppl. Figure 4A. All pathways are significantly downregulated in αCD42b-treated mice relative to IgG-treated mice in Fcgr4⁺ macrophage cluster. In **A** and **B**, p-value < 0.05 and |log2 fold change| ≥0.6, were used.

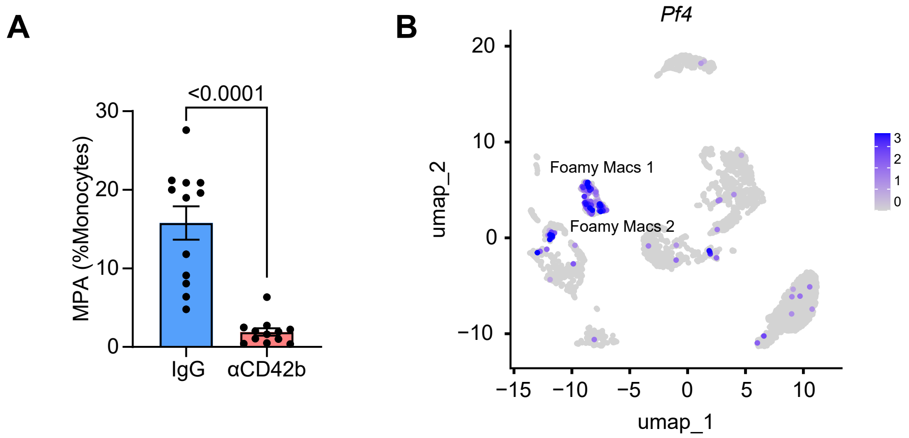

C

Cytokine	Padj	log2FC (αCD42b/IgG)
FGF2	2.85E-07	-0.402250265
BDNF	1.23E-05	-0.331389069
IL6	0.000239	-0.232564877
TGFB1	0.005852	-0.230589526
EGF	0.002442	-0.218562428
NO	0.001993	-0.215686195
HGF	0.043679	-0.150737347
CXCL12	0.04853	-0.140752572
TNFA	0.033922	-0.111731733

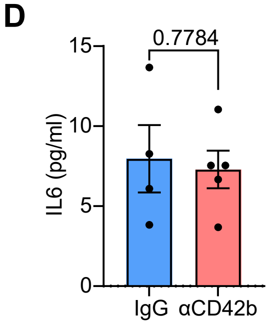

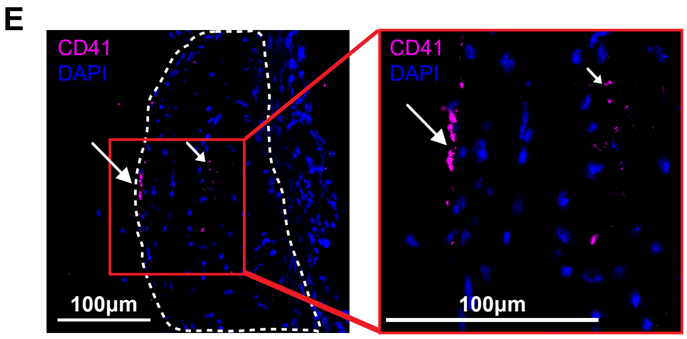

Supplemental Figure 6. Possible mechanisms for the effects of platelets on plaque myeloid cells after lipid-lowering. (A) Monocyte-Platelet aggregates in circulation of IgG- and α CD42b-treated mice. **(B)** UMAP of *Pf4*$^{+}$ cells in CD45^{+} sequenced plaque cells. **(C)** Platelet-related upstream mediators in aCD42 compared to control IgG-treated mice. **(D)** Plasma IL6 levels from IgG- and αCD42b-treated mice. **(E)** CD41^{+} staining in atherosclerotic plaques from obese baseline mice. The arrow at the left points to the EC layer; that on the right is inside the plaque. Data in **D** were analyzed by unpaired Student t-test.

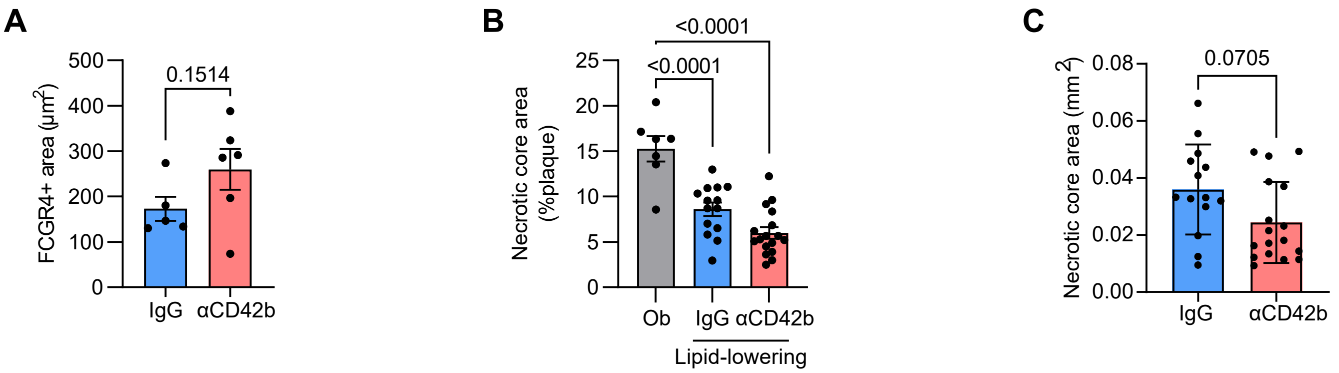

Supplemental Figure 7. During lipid-lowering, platelet depletion leads to increased abundance of FCGR4+ macrophages and reduces necrotic core content in plaques. (**A**) Quantification of FCGR4^{+} area in aortic root plaques of IgG- and αCD42b-treated mice. (**B**) Necrotic core content (%) of Ob, IgG- and αCD42b-treated mice. (**C**) Quantification of necrotic core areas in aortic root plaques of IgG- and αCD42b-treated mice. Data in **A** and **B** were analyzed by unpaired Student t-test or One-way ANOVA Sidak's post hoc test, respectively. Data in **C** were analyzed by unpaired Mann Whitney. p-values shown in graphs.

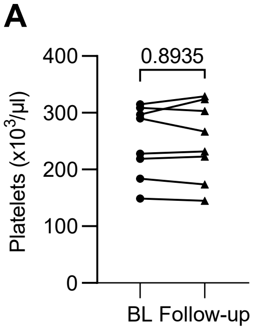

Supplemental Figure 8. Lipid-lowering treatments do not affect platelet counts in individuals with T2D. (**A**) Circulating platelet counts at baseline and follow-up in individuals with T2D from the CHORD study (n=8). Data in **A** were analyzed by a paired Student's t-test.

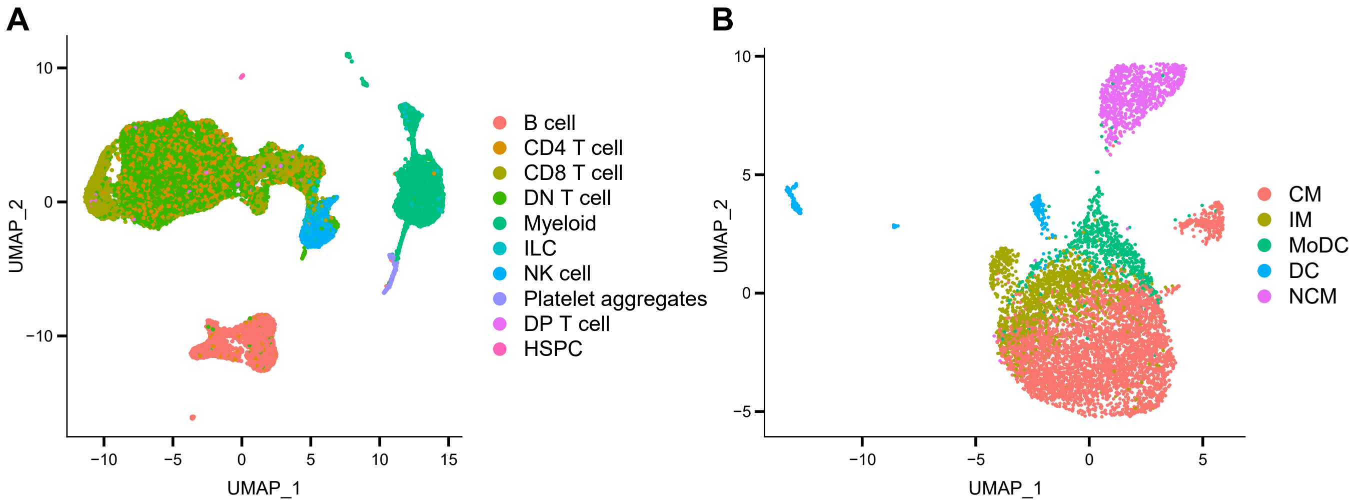

Supplemental Figure 9. PBMC scRNA-seq from subjects enrolled in the CHORD study. (**A**) UMAP of sequenced PBMCs from individuals enrolled in the CHORD study (n=6). (**B**) UMAP of sub-clustered myeloid cells from sequenced PBMCs from individuals, enrolled in the CHORD study (n=6).
